# Pathogenic *SGMS2* variants are not a common cause of early-onset osteoporosis among Finnish patients

**DOI:** 10.3389/fendo.2026.1719375

**Published:** 2026-02-25

**Authors:** Petra Loid, Sampo Richardt, Tuukka Niinimäki, Minna Pekkinen, Outi Mäkitie, Riikka Mäkitie

**Affiliations:** 1 Research Program for Clinical and Molecular Metabolism, Faculty of Medicine, University of Helsinki, Helsinki, Finland; 2 Genetics Research Program, Folkhälsan Research Center, Helsinki, Finland; 3 Children´s Hospital, University of Helsinki and Helsinki University Hospital, Helsinki, Finland; 4 Department of Molecular Medicine and Surgery and Center for Molecular Medicine, Karolinska Institutet, Stockholm, Sweden; 5 Research Unit of Clinical Medicine, University of Oulu, Oulu, Finland; 6 Department of Surgery, Oulu University Hospital, Oulu, Finland; 7 Clinical Genetics, Karolinska University Hospital, Stockholm, Sweden; 8 Department of Otorhinolaryngology - Head and Neck Surgery, Helsinki University Hospital and University of Helsinki, Helsinki, Finland

**Keywords:** early-onset osteoporosis, monogenic osteoporosis, sequencing, SGMS2, single nucleotide variants

## Abstract

**Background:**

Primary osteoporosis can be caused by pathogenic variants in multiple genes. Recently, rare heterozygous variants in *SGMS2*, encoding *SGMS2*, have been identified to cause early-onset osteoporosis or more severe skeletal dysplasia. The incidence of pathogenic *SGMS2* variants and their consequent clinical features, however, remain limited.

**Methods:**

This study aimed to identify the prevalence and nature of *SGMS2* variants in Finnish patients with genetically undiagnosed idiopathic early-onset osteoporosis. All eleven exons and exon-intron boundaries of *SGMS2* were sequenced.

**Results:**

In a cohort of 44 patients (42 females and two males, median age at the time of recruitment 60 years, range 25–76 years), we identified one rare heterozygous missense variant (c.715T>C, p.Phe239Leu) and three intronic variants with unknown functional consequences; no pathogenic or likely pathogenic variants were found.

**Conclusion:**

Our results suggest that pathogenic variants in *SGMS2* are not a common cause of early-onset idiopathic osteoporosis in Finnish patients. Further studies in larger cohorts and variable skeletal phenotypes are needed to increase our understanding of the role of *SGMS2* in skeletal fragility.

## 1 Introduction

Osteoporosis is characterized by reduced bone strength and increased fracture risk with associated increased morbidity and overall healthcare burden (1). Bone health and risk of osteoporosis are highly heritable and usually associated with adverse polygenic traits (2). However, primary osteoporosis may result from pathogenic changes in a single gene, most common ones being genes encoding type I collagen (*COL1A1* and *COL1A2*), or constituents in the WNT (*WNT1* and *LRP5*) or PLS3 signaling pathways (3–6). Advances in genetic studies and our growing understanding of the molecular bases underlying bone metabolism have brought forth new genetic factors contributing to skeletal pathologies (7, 8).

*SGMS2*, encoding the sphingomyelin synthesizing enzyme sphingomyelin synthase 2 (SMS2), is one of the most newly identified genes linked to primary osteoporosis (9). Sphingolipids are important in various cell processes such as cell growth, differentiation, inflammation and apoptosis (10). In 2019, rare heterozygous variants in *SGMS2* were identified in patients presenting with osteoporosis and so called calvarial ‘doughnut’ lesions (OMIM 126550) (9). In several unrelated families early-onset osteoporosis was due to the heterozygous Arg50* variant, suggesting that this is a mutational hotspot in *SGMS2*. Affected patients portray variable degree of skeletal fragility ranging from early-onset osteoporosis in childhood or in early adulthood to more severe spondylometaphyseal dysplasia, and multiple doughnut-shaped sclerotic skull lesions. Other potential clinical features include recurrent cranial nerve palsies, migraine, ataxia, and psychiatric problems, present in a subset of patients (9, 11–13). Although functional, bone biopsy, and zebrafish studies suggest that SMS2 has a role in bone matrix organization and mineralization and that SMS2 is important for normal bone metabolism, the exact mechanisms by which SMS2 regulates bone health remain unclear (9, 14–18).

To date, only 35 individuals with pathogenic *SGMS2* variants have been described in literature. Considering the great variability in their skeletal phenotype (9, 11–13, 19), screening for *SGMS2* may be overlooked in genetically undiagnosed patients with primary osteoporosis. This prompted us to evaluate the prevalence and characteristics of pathogenic *SGMS2* variants in Finnish patients with idiopathic osteoporosis.

## 2 Methods

### 2.1 Study subjects

This study was carried out in collaboration between the Oulu University Hospital, Helsinki University Hospital and Folkhälsan Research Center in Helsinki, Finland. Written informed consent was obtained from all participants and the study was approved by the research ethics committee of the Hospital District of Helsinki and Uusimaa.

The cohort comprised patients with primary osteoporosis of unknown etiology. Clinical data for each individual, consisting of prior fractures, DXA measurements, treatments for osteoporosis and family history of skeletal fragility were collected from electronic patient records and by interview, and carefully reassessed. All patients had BMD measurements taken using dual-X-ray absorptiometry (DXA) from the lumbar spine and/or femoral neck.

We included all individuals with early-onset osteoporosis, defined as osteoporosis diagnosed either premenopausally in women or before age 55 years in men. The inclusion criteria included either 1) an osteoporotic BMD, determined as: T-score ≤ -2.5, combined with a clinically significant fracture history, or 2) low-energy vertebral compression fracture even in the presence of normal BMD Z-score (3, 20). Secondary osteoporosis was excluded in all patients prior to study participation. The patients had previously been screened for *WNT1* variants, with no pathogenic/likely pathogenic variants detected.

### 2.2 Genetic analysis

Genomic DNA was isolated from peripheral blood samples using the Blood DNA Isolation Kit GEB100 (Geneaid, Taiwan). Primers for PCR were designed using Primer3 software and PCR was performed using DreamTaq™ DNA Polymerase (Thermo Fisher Scientific) according to standard protocol. Chromatograms were analyzed with Sequencer v5.3 software (Gene Codes Corporation, USA). Primer sequences are available upon request. All eleven exons and exon-intron boundaries of *SGMS2* were sequenced. Single nucleotide variants (SNVs) and small insertions and deletions were analyzed. The allele frequencies of the identified variants were compared to those obtained from the Genome Aggregation Database (http://gnomad.broadinstitute.org) and the Sequencing Initiative Suomi project (SISu) (https://sisuproject.fi). The potential pathogenicity of the sequence variants was evaluated using different variant prediction databases (SIFT, MutationTaster2, Combined Annotation Dependent Depletion (CADD), MVP, Provean, PrimateAI, Alphamissemse, REVEL, M-CAP and MetaRNN). The HOPE tool (https://www3.cmbi.umcn.nl/hope) was used to predict the structural effect of amino acid change on protein conformation. Splice AI and Pangolin were used to predict splicing effects of the identified variants (21, 22). Proteinpaint (23) was used to visualize the locations of the variants.

## 3 Results

### 3.1 Characteristics of the Finnish cohort with primary osteoporosis

The cohort included 44 patients; 42 females and two males with a mean age of 60 years (range 25–76 years) at the time of recruitment. Their clinical characteristics are presented in **Table 1**. Altogether 35 (80%) of them had a BMD T-score ≤ -2.5 and 17 patients (39%) had a family history of osteoporosis or hip fractures in at least one family member. In total 36 (82%) had a prior or ongoing treatment with osteoporosis medication. Long-bone fractures were found in 35 (80%) patients; 21 had fractures in lower limbs and 14 in upper limbs. Compression fractures were observed in 11 (25%) patients. Over half of the patients had a history of two or more fractures.

**Table 1:** Clinical characteristics of the study cohort with idiopathic osteoporosis.

Number of patients, n	44
Females, n (%)	42(95)
Age, mean (range; years)	60 (25-76)
Patients with osteoporotic BMD, n (%)	35 (80)
Patients with a positive family history, n (%) *	17 (39)
Patients with history of osteoporosis medication, n (%)	36 (82)
Patients with long bone fractures, n (%)	35 (80)
Lower limbs, n (%)	21 (48)
Upper limbs, n (%)	14 (32)
Patients with compression fractures, n (%)	11 (25)
Number of fractures n (%) #	
0	1 (2)
1	18 (41)
2	14 (32)
3	4 (9)
4	1 (2)
“Several”	6 (14)

*Family history includes osteoporosis or hip fractures in at least one family member.

#The number of fractures in “several” was not specified in patient records

### 3.2 Genetic results

**Figure 1:**
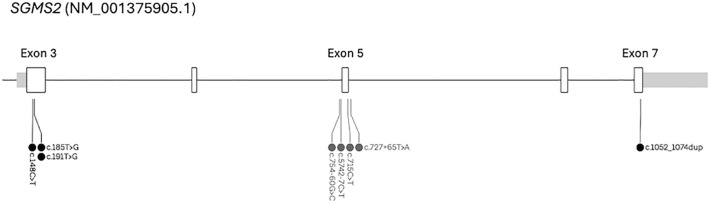
Location of the *SGMS2* variants identified in our study (c.754-60G>C, c.5742-7C>T, c.715C>T, c.727 + 65T>A) and the four previously identified pathogenic *SGMS2* variants (c.148C>T, c.185T>G, c.191T>G, c.1052_1074dup).

Sequencing of the *SGMS2* gene in the 44 patients revealed in none of the patients any of the known pathogenic *SGMS2* variants, including the Arg50* variant which has previously been reported in multiple families. We identified one exonic variant in one patient and three intronic variants in eight patients (**Table 2**). **Figure 1** presents the genomic positions of the *SGMS2* variants.

**Table 2:** Genetic variants in the *SGMS2* gene identified in 44 patients with idiopathic osteoporosis.

Patients (n)	1	6	1	1
Position in gene	exonic	intronic	intronic	intronic
Variant	c.715T>C, p.Phe239Leu	c.574-60G>C	c.574-27C>T	c.727 + 65T>A
rs number	rs200484785	rs9998675	rs138688073	rs1461054400
MAF osteoporosis cohort	0.011	0.064	0.011	0.011
MAF gnomAD	0.0005	0.059	0.012	0
MAF gnomAD FIN	0.0053	0.029	0.029	0
CADD score	26.2	4.4479	1.761	2.377
Splice AI AG (relative position in bp)	0.00 (266)	0.00 (-56)	0.00 (27)	0.00 (189)
Splice AI AL (relative position in bp)	0.00 (10)	0.00 (60)	0.00 (-89)	0.00 (-218)
Splice AI DG (relative position in bp)	0.00 (12)	0.00 (-28)	0.00 (-362)	0.00 (-167)
Splice AI DL (relative position in bp)	0.00 (-25)	0.00 (-329)	0.00 (310)	0.00 (-65)
Pangolin Splice Loss	0.00	0.00	0.00	0.00
Pangolin Splice Gain	0.00	0.00	0.01	0.00

MAF, minor allele frequency.

FIN, Finnish population.

AG, Acceptor Gain.

AL, Acceptor Loss.

DG, Donor Gain.

DL, Donor Loss.

The heterozygous missense variant *SGMS2*(NM_001375905.1): c.715T>C, p.Phe239Leu (rs200484785) was identified in a 69-year-old female patient with BMD T-score -3.5. This variant is enriched in the Finnish population in gnomAD with an allele frequency of 0.0053 (133 heterozygotes) vs 0.0005 in all population in gnomAD. The missense variant is predicted pathogenic/damaging by MutationTaster, Provean, PrimateAI and M-CAP, uncertain by REVEL (0.598) and tolerated/benign by SIFT and MVP. Its CADD score is 26.2, Alphamissense prediction likely pathogenic (score 0.98), while MetaRNN score is 0.01668 (strong benign). This variant affects a highly conserved residue (PhyloP 100way score 8.042). Analysis of the mutant protein by HOPE indicated that the mutant residue is smaller than the wild-type residue and located in the PAP2 superfamily C-terminal domain that is important for the main activity of the protein and in a highly conserved region. This variant has been reported as benign in ClinVar (1 submission,1 star).

In addition, three heterozygous intronic variants were identified in eight patients: c.574-60G>C (rs9998675) in six patients, c.574-27C>T (rs138688073) in one patient and c.727 + 65T>A (rs1461054400) in one patient. The variants rs9998675 and rs138688073 have an allele frequency of 0.029 in the Finnish population in gnomAD and the variant rs1461054400 is not found in gnomAD or SISu. Splice AI and Pangolin predicted no effect on splicing for these variants (**Table 2**).

## 4 Discussion

The *SGMS2* gene was recently identified, by us and since others, as a key regulator of skeletal health and its pathogenic variants to result in severe primary osteoporosis (9). As the clinical phenotype is highly variable and much of the molecular mechanisms underlying the associated skeletal fragility still unknown, the prevalence and genetic characteristics of SGMS2-related osteoporosis are still to be determined. Our study identified four variants in nine of 44 patients with primary osteoporosis; none were considered pathogenic or likely pathogenic while their true genetic significance remains to be determined. Importantly, the known pathogenic variants, including the hotspot variant Arg50*, were not observed. Taken together, *SGMS2*-related skeletal fragility remains rare and pathogenic gene variants an uncommon cause of primary osteoporosis. While our results suggest that the gene’s disease-causing variants in patients with osteoporosis cluster primarily in exon 3, the full spectrum and function of allele variance demand further evaluation.

As confirmed by our findings, pathogenic variants in *SGMS2* remain rare and are unlikely to underlie milder forms of primary osteoporosis. To date, four different pathogenic *SGMS2* variants have been identified in patients with variable skeletal phenotypes: a nonsense variant (c.148C>T, p.Arg50*), two missense variants (c.185T>G, p.Ile62Ser and c.191T>G, p.Met64Arg), and a frameshift variant (c.1052_1074dup, p.Glu359Ilefs*49) (9, 11–13, 19). Whereas the nonsense variant results in childhood-onset osteoporosis with low BMD, skeletal fragility, and multiple fractures, the two missense variants associate with a more severe phenotype with spondylometaphyseal dysplasia with long-bone deformities, neonatal fractures, and short stature (9). The frameshift variant c.1052_1074dup was recently reported in a family with recurrent fractures but high bone mass (19). In a cohort of 44 patients with early-onset osteoporosis we found no novel variants and four rare *SGMS2* variants in nine patients: one missense variant and three intronic variants, none of which were clearly pathogenic. The heterozygous missense variant p.Phe239Leu is enriched in the Finnish population and the *in silico* prediction tools showed variable predictions, and therefore its clinical significance remains unclear. The three intronic variants (rs9998675, rs138688073 and rs1461054400) have unknown functional consequences with very limited information available.

The exact mechanisms by how pathogenic *SGMS2* variants relay to a skeletal pathology remain incompletely understood. Previous studies have shown that the nonsense variant p.Arg50* results in a catalytically inactive enzyme and the two missense variants p.Ile62Ser and p.Met64Arg cause retention of SMS2 to the ER, producing sphingomyelin in the wrong place (9, 18). Furthermore, analysis of bone biopsy of two patients with bone fragility harboring *SGMS2* variant p.Arg50* showed low bone matrix mineralization, disorganized collagenous fibrils and disturbed osteocyte lacunae and lacuna-canalicular network (15). A recent transcriptomics and lipidomics study on patient-derived fibroblasts suggests that SGMS2 variants may influence the circadian rhythm and gap junction formation, which could negatively affect bone health and homeostasis (16). Taken together, the disruption in normal SMS2 signaling results in evident disturbance of normal bone metabolism. The molecular details and factors contributing to individual heterogeneity in the subsequent skeletal outcome are still, however, unknown.

Furthermore, the spectrum of clinical features associated with aberrant SMS2 signaling is also unclear. Prior studies have outlined that clinical manifestations can be highly variable even within a family or have incomplete penetrance (13). Furthermore, next to the skeletal phenotype, *SGSM2* variants also associate with a multitude of extra-skeletal traits. Identified patients portray a spectrum of various neurological characteristics, most common ones being transient and recurrent facial and other cranial nerve palsies, ataxia, migraines, and psychiatric problems. These may suggest that, instead of isolated primary osteoporosis, *SGMS2* variants result in a more systemic and syndromic phenotype, which would also support the ubiquitous role of sphingolipids in multiple organs and their broad functions in developmental and homeostatic processes. However, considering the range in severity of *SGMS2* osteoporosis, milder phenotypes and in the absence of extra-skeletal features should not be overlooked and *SGMS2* carefully considered.

We recognize some limitations in our study including the relatively small size of the study cohort, somewhat limited clinical information available from each patient and the absence of a control group. Detailed clinical data on fracture characteristics and in-depth bone quality assessments would better outline the patients’ skeletal phenotypes and possible relevance of identified variants. Furthermore, although the identified variants were compared against informative databases, the Finnish population in gnomAD and SISu project, and analyzed by various web-based computational platforms, the lack of functional studies limits drawing further conclusions about the potential functional consequences of the identified variants. Despite these, we consider our study to offer valuable information about the significance of *SGMS2* in primary osteoporosis. Our findings provide important prevalence data from a well-characterized cohort of patients with idiopathic early-onset osteoporosis and indicate that routine SGMS2 screening may have limited diagnostic utility in milder forms of primary osteoporosis.

In conclusion, our findings indicate that pathogenic *SGMS2* variants are not a common cause of primary osteoporosis among Finnish patients. More studies in larger cohorts, other disease groups, and in conjunction with in-depth clinical analyses, including e.g., more detailed characterization of skeletal pathology, fracture healing, surgical outcomes and response to anti-resorptive and anabolic therapies, are needed to further determine the prevalence of *SGMS2* variants and increase our understanding the role of *SGMS2* variants in human disease.

## Data Availability

The original contributions presented in the study are included in the article/supplementary material. Further inquiries can be directed to the corresponding author/s.
